# Prognostic factors in squamous cell carcinoma of the oral cavity

**DOI:** 10.1016/S1808-8694(15)30146-4

**Published:** 2015-10-18

**Authors:** José Raphael de Moura Campos Montoro, Hilton Alves Hicz, Luiz de Souza, David Livingstone, Daniel Hardy Melo, Rogério Costa Tiveron, Rui Celso M Mamede

**Affiliations:** a Master”s degree student, assistant physician in Head & Neck Surgery, Otorhinolaryngology Unit, Hospital do Servidor Público Estadual de São Paulo. Graduate student, Ophthalmology, Otorhinolaryngology and Head & Neck Surgery Department, Hospital das Clínicas da Faculdade de Medicina de Ribeirão Preto; b Doctoral degree, assistant professor, Head & Neck Discipline, Hospital das Clínicas da Faculdade de Medicina de Ribeirão Preto; c Doctoral degree, assistant professor, Puericultura and Pediatrics, Department, Faculdade de Medicina de Ribeirão Preto - USP; d Master”s degree. Doctoral student, graduate student, Ophthalmology, Otorhinolaryngology and Head & Neck Surgery Department, Hospital das Clínicas da Faculdade de Medicina de Ribeirão Preto; e Master”s degree student, graduate student Ophthalmology, Otorhinolaryngology and Head & Neck Surgery Department, Hospital das Clínicas da Faculdade de Medicina de Ribeirão Preto; f Doctoral student, Ophthalmology, Otorhinolaryngology and Head & Neck Surgery Department, Hospital das Clínicas da Faculdade de Medicina de Ribeirão Preto; g Doctoral degree, livre-docente (habilitation), full professor, Head & Neck Surgery Discipline Hospital, Clínicas da Faculdade de Medicina de Ribeirão Preto; h Hospital das Clínicas da Faculdade de Medicina de Ribeirão Preto - USP

**Keywords:** prognosis, carcinoma, oral cancer, squamous cell

## Abstract

Researchers have been looking for factors that can influence the prognosis of oral cancer, because its outcome is highly uncertain. **Aim**: To evaluate variables that can impact the survival rate of patients with squamous-cell carcinoma of the oral cavity. **Material and Methods**: Data analysis of 45 patients from January, 2001 to January, 2006. Survival rate curves have been estimated using the Kaplan-Meier method and they have been compared through the log-rank test and the Cox regression standard. Study design: Retrospective analysis. **Results**: Total five-year survival rate was of 39% fpr these patients. Only the neck metastases (p=0.017), postoperative radiotherapy (p=0.056) and diseased margin(p=0.004) variables had statistic relevance. Survival rate was lower in patients with neck metastases, margins involved and those who underwent postoperative radiotherapy, in other words, those with the most aggressive tumors. After adjustment, radiotherapy did not prove to be statistically relevant. It is likely that the survival rate of 39% was due to the high number of patients with metastasis (52%) and because the samples were mostly of tongue and mouth floor diseases (82%), which are the hardest to control. **Conclusion**: Neck metastases and diseased margins of oral cavity carcinomas are the prognostic factors that can most impact the survival rate.

## INTRODUCTION

According to the World Health Organization (WHO), mouth and oropharyngeal cancers are the most frequent head and neck malignancies; 390 thousand new cases are reported every year.^1^ In Brazil, mouth and oropharyngeal cancers are the fifth most frequent malignancies among males (9.2% of cases) and the seventh most frequent in females (3.6% of cases) not including skin cancers.^2^ In total these malignancies are 6.7% of all cancer cases. These malignancies occur most frequently in south and southeast Brazil.^2^, ^3^ Estimates for 2007 had suggested that there would be 10.91 new cases or mouth cancer for each 100,000 males and 3.58 new cases for each 100,000 females.^4^ The incidence rate is increasing and the WHO foresees further increase in the next decades.^5^ Around 95% of mouth cancers are squamous cell carcinomas (SCC); the remaining 5% are sarcomas, lymphomas and salivary gland tumors.

The prognosis of oral cancer remains unfavorable with high mortality rates, notwithstanding advances in diagnosis and therapy, including radical surgery, novel chemotherapy, and hyperfractionated/conformational radiotherapy. Mortality rates have ranged from 2.16 to 2.96 for each 100,000 males and 0.48 and 0.70 for each 100,000 females^6^ between 1979 and 1998 in Brazil; the mortality rate has increased at an annual 0.72% rate.7 According to Sessions et al., the 5-year survival rate remains low, at about 48% (overall survival) and 57% (disease-specific survival).^8^

The biological behavior of oral SCC is uncertain; many of these tumors have an aggressive biological behavior at initial stages with early regional metastases and death. On the other hand, advanced tumors may metastasize slowly, and these patients may remain disease-free for long periods after surgery.

This uncertainty in tumor progression has led researchers to seek factors that might alter the prognosis. Such factors may be related to patients (age, sex, race, social and economic status, and habits such as smoking and alcohol intake), to the tumor (site, stage, tumor thickness, histopathology, and expression of certain molecular markers), and to the treatment (type of treatment, adjuvant therapy). Investigation of these factors aims to learn more about the biological behavior of the tumor, so that specific strategies may be applied individually; thus aggressive therapy may be given to patients with the worst prognosis.

The presence of neck metastases is the most important prognostic factor for oral SCC; if present, there is a 50% reduction in survival rates.^9^, ^10^ TNM staging, the histological grade and safety margins are other factors with unknown roles. Some studies have suggested that TNM staging (a tool used for establishing a prognosis) cannot predict individual tumor biological behavior. The prognostic value of the histological grade is controversial in this tumor;11 studies have suggested that poorly differentiated carcinomas tend to metastasize and to have involved margins more often. These tumors are associated with decreased survival rates.^12^ There is still controversy in the literature about margin status.

The purpose of this paper was to evaluate variables related to patient, tumor and treatment variables affecting survival rates in mouth SCC.

## MATERIAL AND METHOD

### Patients

Information about 45 patients with primary mouth SCC, between January 2001 and January 2006, from the “Head & Neck Surgical Sample Bank” was reviewed. The Research Ethics Committee approved this study (protocol number 9371/2003). Data was collected about patients (age, sex, smoking and alcohol intake), the tumor (site, TNM stage, degree of differentiation, vascular, lymphatic and perineural spread, inflammation around the tumor), treatment (margin status, postoperative radiotherapy), and progression. We were unable to separate patients that died due to the tumor or due to other causes; thus, only overall survival was calculated, rather than the disease-specific survival.

Patients with primary mouth SCC treated initially with curative surgery at our institution, from which biological material was biopsied during surgery and stored, with follow-up and documented histological data kept at the database of the Genome Project, were included. Patients with lip tumors and patients with no follow-up were excluded.

### Diagnosis and staging

Tumor diagnosis was based on a clinical examination followed by a biopsy of the lesion and pathology. Computed tomography was done to assess tumor spread and the presence of cervical nodes. All patients were staged according to the International Union Against Cancer (UICC) 2002 guidelines.

### Treatment and Follow-up

All patients underwent surgical removal with safety margins of the primary lesions with a curative intent. Radical neck dissection was done for removing neck metastases identified clinically or radiologically; elective neck dissection was done in T2 to T4-staged patients with no neck metastases. Patients with T4-staged tumors or in whom histopathology revealed involved margins, angiolymphatic or perineural spread, or lymph node metastases underwent postoperative radiotherapy. Following the treatment patients returned on the 1st, 3rd, 6th and 12th months in the first year and thereafter every 6 months.

### Statistical analysis

Survival curves for each variable were estimated using the Kaplan-Meier method; the log-rank test was used for comparison purposes. The Cox regression model was used for checking the effect of each variable after each was adjusted to the same level (Hosmer & Lemeshow, 1999).^13^

## RESULTS

### Description of the sample

Table 1 shows the frequency distribution for each level of each variable according to the vital status of patients. The five levels of the subsite variable were grouped into three levels: floor of the mouth, tongue, and other (gingival, n=5; palate, n=1; and retromolar, n=2) to make the survival analysis feasible. The poorly differentiated group contained only two patients, and was therefore added to the group of moderately differentiated tumor group. The mean age was 56.3 years, the standard deviation was 10.3 years; the age percentages were as follows: P25=52 years, P50(median)=55 years, and P75=63 years. Fifty percent of patients were aged between 55 and 63 years. Age was dichotomized and limited to 60 years in the survival analysis.

**Table 1:** Distribution of variable frequencies in 45 operated patients with mouth cancer between 2001 and 2006, according to the vital status.

Variable		Death		Non death		Total
		n	%	n	%	
Sex	Male	22	55,0	18	45,0	40
Age	Female	2	40,0	3	60,0	5
Smoking	³60 years	7	46,7	8	53,3	15
Alcohol use	< 60 years	17	56,7	13	43,3	30
Subsite	Yes	21	53,9	18	46,1	39
T staging	No	3	50,0	3	50,0	6
N staging	Yes	18	58,1	13	41,9	31
Postoperative radiotherapy	No	6	42,9	8	57,1	14
Differentiation grade	Floor	10	55,6	8	44,4	18
Vascular spreadr	Gingiva	2	40,0	3	60,0	5
Lymphatic spread	Tongue	11	57,9	8	42,1	19
Perineural spread	Palate	0	0	1	100,0	1
Peritumor inflammation	Retromolar	1	50,0	1	50,0	2
Margins	T1	4	50,0	4	50,0	8
	T2	12	54,6	10	46,4	22
	T3	5	55,6	4	44,4	9
	T4	3	50,0	3	50,0	6
	N	17	65,4	9	34,6	26
	N-	7	36,8	12	63,2	19
	Yes	14	63,6	8	36,4	22
	No	10	43,5	13	56,5	23
	Well differentiated	11	47,8	12	52,2	23
	Moderately diff	11	55,0	9	45,0	20
	Poorly differentiated	2	100,0	0	0	2
	Presence	4	44,4	5	55,6	9
	Absence	20	55,6	16	44,4	36
	Presence	5	55,6	4	44,4	9
	Absence	19	52,8	17	47,2	36
	Presence	4	36,4	7	63,6	11
	Absence	20	58,8	14	41,2	34
	Intense	1	20,0	4	80,0	5
	Moderate	6	66,7	3	33,3	9
	Absent	17	54,8	14	45,2	31
	Compromised	11	78,6	3	21,4	14
	Free	12	40,0	18	60,0	30
	Not assessed	1	100,0	0	0	1

Total		24	53,3	21	46,7	45

Answers on the variables sex and smoking were concentrated in only one level, as follows: 40 (88.9%) of patients were male and 39 (86,7%) patients were smokers. There was thus less assurance in the test results involving these two variables.

### Estimated survival curves

**Figure 1:**
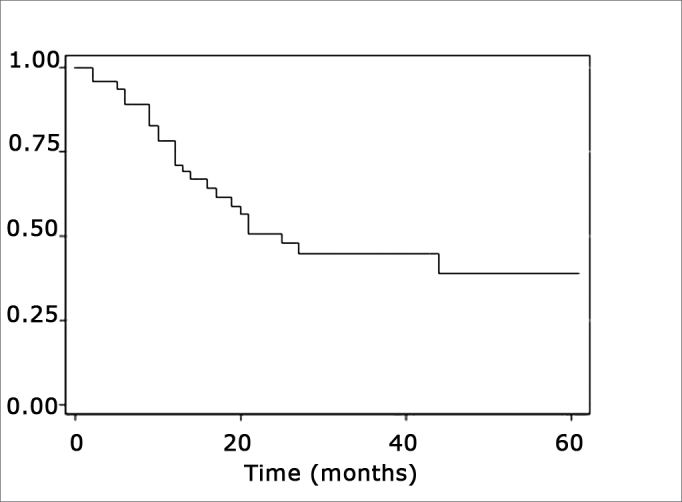
Representation of the survival curve of patients with mouth cancer, estimated by the Kaplan-Meier method.

The estimated overall survival curve by the Kaplan-Meier method and the 5-year survival rate was 39% (Fig. 1).

**Figure 2:**
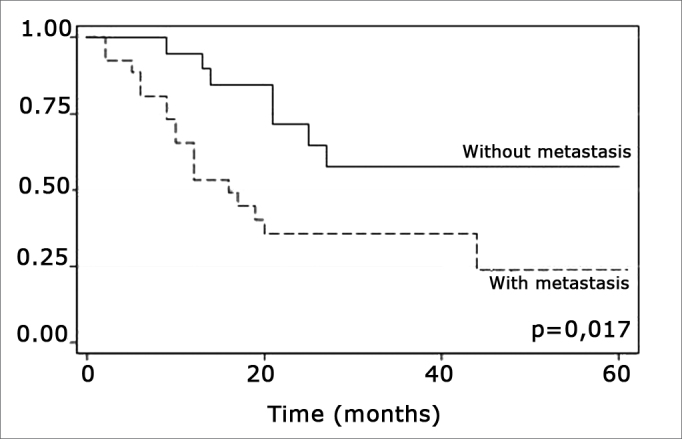
Representation of survival curves for the presence and absence of neck metastases, estimated by the Kaplan-Meier method (p=0.017, according to the log-rank).

**Figure 3:**
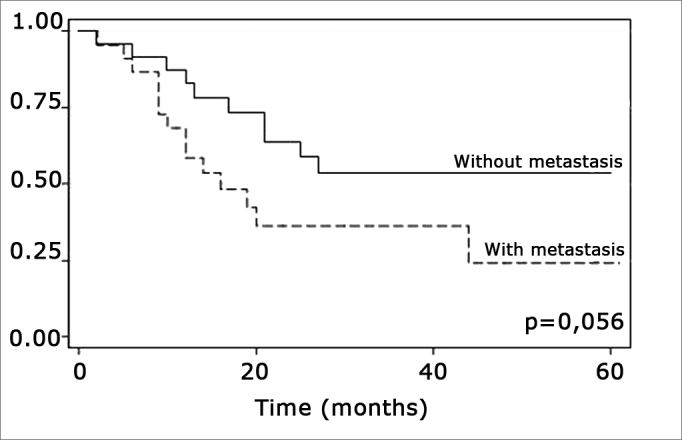
Representation of survival curves for postoperative radiotherapy and its absence, estimated by the Kaplan-Meier method (p=0.056, according to the log-rank).

**Figure 4:**
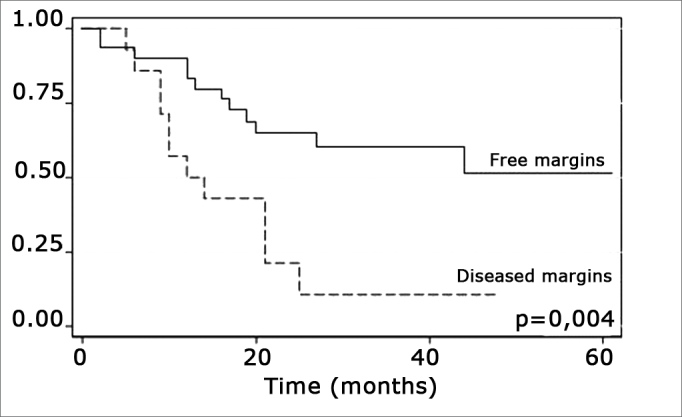
Representation of survival curves for free and compromised margins, estimated by the Kaplan-Meier method (p=0.004, according to the log-rank).

The log-rank test, which tests the effect of each variable singly on survival, revealed the following significant factors: neck metastasis (p=0.017), postoperative radiotherapy (p=0.056) and involved margins (p=0.004). The other variables had p values equal to or over 0.24. Figs. 2, 3 and 4 show the estimated survival probabilities and curves for the abovementioned three variables. For instance, Fig. 2 shows that the estimated probability of surviving more than 9 months was 95% (CI-95%: 68%-99%) for metastasis-free patients, and 73% (CI-95%: 52%-86%) for patients with metastases. Survival was lower in patients with neck metastases that underwent postoperative radiotherapy and who had involved margins (Figs. 3 and 4). The results of radiotherapy seem contradictory, as one would expect increased survival when using this treatment. Eighteen (81.8%) of 22 patients undergoing radiotherapy had neck metastases, in other words, patients with more aggressive tumors.

### Effect adjusted

The variables metastasis, radiotherapy and margins were considered jointly in the Cox regression model to verify the effect of each variable and their relation with other in the same level. Radiotherapy was not significant (p=0.816), while metastasis (p=0.002) and margins (p<0.001) were relevant factors for patient survival times.

## DISCUSSION

Mouth SCCs have a poor prognosis and tend to recur in the primary site and the neck. The 5-year survival rate in our sample was 39%, which is lower than other published results. This is probably due to the higher presence of patients with metastatic lymph nodes (52.2%) and the fact that the sample consisted mostly of tongue and floor of mouth cancers (82%), which are recognizably more difficult to control. These values differ from Sessions et al.”s series in which only 26% of patients had metastatic lymph nodes.8 Furthermore, our sample contained 34.8% of T3/T4 tumors; this percentage in Sessions et al.”s series was 26.5%.8 Additionally the biological behavior of mouth SCCs is heterogeneous, depending of poorly known host and primary tumor factors. Figueiredo et al. have previously published data on the first 15 mouth tumors in our present series, showing that these patients expressed highly the PRAME gene; this finding is related with the presence of lymphatic metastases.^14^

We detected no patient-related factor among those we studied that had any effect on survival; age, sex, smoking and alcohol use had no statistically significant effect. There is no consensus in the literature about the influence of these factors; according to EIBAND, there is no correlation with age, sex or the prognosis,^15^ while smoking and alcohol use are seen as risk rather than prognostic factors.

Tumor-related factors (subsite, size, tumor differentiation, peritumor inflammation, and vascular, lymphatic and perineural spread) had no statistical significance. Kademani et al. have shown that the tumor site has not effect on survival; these authors did suggest that the histological grade was a statistically significant prognostic factor.^12^ The histological grade appears not to have affected survival due to the limited number of cases of undifferentiated carcinomas in our sample (two cases). There is no consensus in the literature about whether vascular, lymphatic and perineural spread decrease survival.^16^ The sensitivity of the method for detecting spread (immunohistochemistry is much more sensitive than hematoxyllin-eosin) and the expertise of the pathologist may explain these conflicting conclusions.

Neck metastasis was the only tumor-related factor that has been shown to decrease survival in this sample (p=0.017), confirming most of the corresponding published results.^9^, ^10^ It is relevant to point out that the metastatic rate in our sample of mouth cancers was 57.7%.

Treatment-related factors, such as compromised surgical margins and supplementary radiotherapy, had an impact on survival. Confirmation that margin status has an effect on survival is important, as compromised margins may be avoided by ample surgical resection. According to Mistry et al., however, this is not an easy task; these authors found that measurements of free mucosal margins - attained in surgery - decreased by up to 23% postoperatively, particularly in T1/T2 tumors.^19^ Spiro et al. found an increased local recurrence in patients with involved margins, which did not alter overall survival.^18^

The presence of compromised margins, which was statistically significant (p=0.004) in our sample, agrees with Sessions et al.”s results in showing that compromised margins decreased disease-free survival; these authors also found that when patients with compromised margins underwent postoperative radiotherapy, disease-specific survival increased significantly.^8^ Chao et al. also demonstrated that postoperative radiotherapy provided good results in patients with compromised margins; this group of patients had the same survival and local control that was found in patients with free margins. Postoperative radiotherapy did not improve survival in our sample.^17^ Other authors also did not find the abovementioned significance (Kademi et al.).^12^

The log-rank test showed that postoperative radiotherapy (p=0.056) had an opposite effect, namely that patients undergoing radiotherapy had a lower survival rate. Radiotherapy was not statistically significant (p=0,816), however, when considering radiotherapy, neck metastasis and compromised margins jointly in the Cox regression model; other variables were statistically significant. This may be a result of indication of treatment criteria whereby only more aggressive tumors were treated with radiotherapy postoperatively. Radiotherapy should be indicated carefully - as well as taking into account tumor aggressiveness - as according to Brandwein-Gensler et al., radiotherapy is only useful when applied in cases with a high risk of recurrence (truly compromised margins).^21^

While Ord et al.”s study involved only one surgeon - to decrease added effects^20^ - many surgeons participated in our study, as our institution has a residency program.

Molecular assessment may provide additional information about tumors; thus the Gencapo study group - in counting tumors in many institutions - may in the near future provide relevant data for improving individual treatment.

## CONCLUSION

We may conclude that neck metastases from mouth SCCs (a tumor-related prognostic factor) and surgical margin status (related to therapy) affected the survival of patients.

## References

[1] Stewart BW, Kleihues P (2003). World cancer report.

[2] Wünsch Filho V. (2002). The epidemiology of oral and pharynx cancer in Brazil. Oral Oncol.

[3] Ministério da Saúde; Secretária de Assistência à Saúde (2004). Registro Hospitalar de Câncer: dados dos hospitais do INCA, relatório anual 1994/1998. Distribuição dos casos de câncer por localização topográfica, segundo o sexo.

[4] (2005). Estimativa 2006: incidência de câncer no Brasil.

[5] Bettendorf O, Piffkò J, Bànkfalvi A. (2004). Prognostic and predictive factors in oral squamous cell cancer: important tools for planning individual therapy?. Oral Oncol.

[6] Ministério da Saúde; DATASUS, Instituto Brasileiro de Geografia e Estatística (2002). Instituto Nacional de Câncer. Falando sobre o câncer de boca.

[8] Sessions DG, Spector GJ, Lenox J, Haughey B, Chao C, Marks J (2002). Analysis of treatment results for oral tongue cancer. Laryngoscope.

[9] Shah J (1990). Cervical lymph node metastasis, its diagnostic, therapeutic and prognostic implications. Oncology.

[10] Grandi C, Allossio M, Moglia DEA (1985). Prognostic significance of lymphatic spread in head and neck carcinomas: therapeutic implications. Head Neck Surg.

[12] Kademani D, Bell RB, Bagheri S, Holmgren E, Dierks E, Potter B, Homer L (2005). Prognostic factors in intraoral squamous cell carcinoma: the influence of histologic grade. J Oral Maxillofac Surg.

[13] Hosmer DW, Lemeshow S (1999). Applied Survival Analysis.

[14] Figueiredo DLA, Mamede RCM, Protosiqueira R, Neder W, Zago MA (2006). Expression of cancer testis antigens in head and neck squamous cell carcinomas. Otolaryngol Head Neck Surg.

[15] Eiband JD, Elias EG, Suter CM, Gray WC, Didolkar MS (1989). Prognostic factors in squamous cell carcinoma of the larynx. Am J Surg.

[16] Kurtz KA, Hoffman HT, Zimmerman MB, Robinson RA (2005). Perineural and vascular invasion in oral cavity squamous carcinoma. Arch Pathol Lab Med.

[17] Chao KSC, Emami B, Akhileswaran R, Simpson J, Spector G, Sessions D (1996). The impact of surgical margin status and use of an interstitial implant on T1, T2 oral tongue cancers after surgery. Int J Radiat Oncol Biol Phys.

[18] Spiro RH, Guillamondegui O, Paulino AF, Huvos AG (1999 Aug). Pattern of invasion and margin assessment in patients with oral tongue cancer. Head Neck.

[19] Mistry RC, Qureshi SS, Kumaran C (2005). Post-resection mucosal margin shrinkage in oral cancer: quantification and significance. J Surg Oncol.

[20] Ord RA, Kolokythas A (2006). Reynolds MA: Surgical salvage for local and regional recurrence in oral cancer. J Oral Maxillofac Surg.

[21] Brandwein-Gensler M, Teixeira MS (2005). Oral squamous cell carcinoma: histologic risk assessment, but not margin status, is strongly predictive of local disease-free and overall survival. Am J Surg Pathol.

